# Potential of Preoperative 11C-Methionine Positron Emission Tomography in Predicting EGFR Alterations and CDKN2A/B Homozygous Deletion in Diffuse Astrocytic Gliomas

**DOI:** 10.7759/cureus.87747

**Published:** 2025-07-11

**Authors:** Keisuke Masuda, Nayuta Higa, Toshiaki Akahane, Shingo Baba, Kazutaka Yatsushiro, Hajime Yonezawa, Hiroyuki Uchida, Ryutaro Makino, Tomoko Takajo, Mari Kirishima, Takuro Isoda, Daisuke Kuga, Koji Yoshimoto, Akihide Tanimoto, Ryosuke Hanaya

**Affiliations:** 1 Department of Neurosurgery, Graduate School of Medical and Dental Sciences, Kagoshima University, Kagoshima, JPN; 2 Department of Pathology, Graduate School of Medical and Dental Sciences, Kagoshima University, Kagoshima, JPN; 3 Department of Health Sciences, Graduate School of Medical Sciences, Kyushu University, Fukuoka, JPN; 4 Department of Neurosurgery, Fujimoto General Hospital, Miyakonojo, JPN; 5 Department of Clinical Radiology, Graduate School of Medical Sciences, Kyushu University, Fukuoka, JPN; 6 Department of Neurosurgery, Graduate School of Medical Sciences, Kyushu University, Fukuoka, JPN

**Keywords:** cdkn2a/b, egfr, fluorine-18 fluorodeoxyglucose, glioma, l-methyl-11c-methionine

## Abstract

Objective

L-methyl-^11^C-methionine (MET)- and ^18^F-fluorodeoxyglucose (FDG)-positron emission tomography (PET) are used to detect gliomas. However, the efficacy of MET-PET and FDG-PET in detecting gene alterations in gliomas remains unclear. Therefore, in this study, we evaluated the relationship between genetic alterations and PET tracer uptake in diffuse astrocytic glioma.

Methods

Thirty-two patients who had been newly diagnosed with astrocytic gliomas at Kagoshima University and Kyushu University and had undergone MET-PET and FDG-PET were enrolled. They underwent analysis of glioma-related gene expression using a customized 48-gene panel.

Results

The tumors identified in this study were classified as follows: glioblastomas, isocitrate dehydrogenase (IDH) wildtype (n = 15); astrocytic glioma, IDH-mutant, World Health Organization (WHO) grade 4 (n = 2); astrocytic glioma, IDH-mutant, WHO grade 3 (n = 4); astrocytic glioma, IDH-mutant, WHO grade 2 (n = 7); and diffuse astrocytic glioma, not elsewhere classified (n = 4). Astrocytic tumors with *IDH *mutations, *ATRX *mutations, and/or loss of function (mut/loss) had a significantly lower tumor-to-normal tissue (T/N) ratio on the MET-PET and FDG-PET images compared with those without these alterations. Astrocytic tumors with *CDKN2A/B* homozygous deletions (HD), *EGFR *mutation and/or amplification (mut/amp), or *PTEN* mut/loss had a significantly higher T/N ratio on the MET-PET images compared with those without these alterations. Astrocytic tumors with *NF1* mut/loss had a significantly higher T/N ratio on their FDG-PET images compared with those without these alterations. The cut-off T/N ratio for the MET-PET images for the identification of *EGFR* mut/amp was 4.50 (sensitivity: 95%; specificity: 56%, AUC: 0.77), and that for detecting* CDKN2A/B *HDwas 2.32 (sensitivity: 72%; specificity: 86%; AUC: 0.85).

Conclusion

These findings from our small, retrospective cohort study suggest that MET-PET and FDG-PET are potentially valuable approaches for preoperatively predicting the molecular status of gliomas, particularly for assessing tumors with *EGFR* mut/amp and *CDKN2A/B *HD. Preoperative genetic alteration prediction in astrocytic gliomas based on PET tracer uptake may provide accurate information for patients and inform clinical decision-making. Multicenter prospective trials are essential.

## Introduction

In 2021, the guidance for detailed tumor molecular characterization was incorporated into the fifth edition of the World Health Organization (WHO) Classification of Brain Tumors 2021 [1]. Isocitrate dehydrogenase (IDH)-wildtype adult-type diffuse astrocytic tumors with EGFR amplification, TERT promoter (TERTp) mutations, or combined gain of chromosome 7 and loss of chromosome 10 can be classified as glioblastomas (GBM), even with low histological grades. Furthermore, IDH-mutant adult-type diffuse astrocytic tumors with CDKN2A/B homozygous deletion (HD) can be classified as grade 4 astrocytic glioma, even if the histological grade is low [1]. The aforementioned molecular markers can serve as prognostic factors for gliomas; therefore, their preoperative identification can help determine the surgical approach and treatment strategies.

For example, a preoperative identification of an IDH-wildtype astrocytic glioma would warrant a more thorough resection. Moreover, the advent of IDH inhibitors is providing a foundation for novel neoadjuvant treatment strategies. Perioperative studies, such as that conducted by Cain et al., are underway to investigate the administration of IDH inhibitors to patients with IDH-mutant gliomas prior to surgical resection [2]. Although this is not yet an established practice, the preoperative administration of IDH inhibitors has the potential to become a valuable therapeutic strategy, particularly for patients with IDH-mutant gliomas in eloquent areas.

Positron emission tomography (PET) is a valuable imaging modality for the metabolic characterization of tumors. L-methyl-11C-methionine (MET)-PET and 18F-fluorodeoxyglucose (FDG)-PET are commonly used for brain tumor detection. They complement Ki-67 index staining [3,4] and are utilized for tumor grading [5] and prognosis prediction [6,7]. Based on these metabolic characterization abilities, we hypothesized that assessing metabolic dynamics using PET would enable a more accurate prediction of genetic alterations in gliomas. However, the correlation between genetic alterations in gliomas and PET tracer accumulation has only been investigated using certain genes, including IDH [7], ATRX [8,9], and TERT [10]. Furthermore, the utility of MET-PET imaging in predicting EGFR amplification and CDKN2A/B homozygous deletion has not been studied. In this study, we investigated the correlation between gene alterations in gliomas and MET and FDG accumulation using PET.

## Materials and methods

Patients

Thirty-two patients who had been newly diagnosed with astrocytic gliomas and had undergone MET-PET and FDG-PET before surgery between 2015 and 2021 were included in this study. Biopsy or resection sections from the patients were used for histopathological evaluation. All tumors were subjected to genetic analysis and graded using the WHO 2021 classification for Central Nervous System Tumors.

DNA extraction and quantification

The Maxwell 16 FFPE tissue LEV DNA purification kit (Promega K.K., Tokyo, Japan) was used for DNA extraction from formalin-fixed paraffin-embedded samples, according to the manufacturer’s instructions. Then, DNA concentration was measured using a Qubit 3.0 Fluorometer dsDNA BR assay kit (Thermo Fisher Scientific, Waltham, United States), and the DNA quality was assessed using a QIAseq DNA QuantiMIZE kit (QIAGEN, Hulsterweg, Netherlands). The extracted DNA was diluted to a concentration of 5-10 ng/μL for subsequent amplification using a polymerase chain reaction (QIAseq DNA QuantiMIZE kit).

Next-generation sequencing

Next-generation sequencing (NGS) was performed using an amplicon-based glioma-tailored gene panel as described previously [11]. The 48 genes in the panel are listed in Table 1. These genes were selected to include the most frequently mutated genes in gliomas and key diagnostically relevant markers. Additionally, the panel included genes on chromosome arms 1p and 19q to allow an assessment of 1p/19q codeletion status [11]. Copy number alterations and single-nucleotide variants were evaluated using an NGS onco-panel. The amplicon sequences were aligned to the human reference genome GRCh37 (hg19) in the target sequence region. Data were analyzed using the QIAGEN web portal service (https://www.qiagen.com/).

**Table 1:** List of the 48 genes used in this study

Target Genes
IDH1, IDH2, ATRX, TP53, TERT, NF1, EGFR, RB1, PDGFRA, PTEN, CDK4, CDKN2A/B, BRAF, MDM2, FGFR1, ACVR1, HIST1H3B, HIST1H3C, MAP2K4, CDK12, ATM, CCND1, CDK6
Chromosome 1
TNFRSF14 (1p36.32), MTOR (1p36.22), SDHB (1p36.13), ARID1A (1p36.11), MUTYH (1p34.1), JAK1 (1p31.3), FUBP1 (1p31.1), FAM46C (1p12), NOTCH2 (1p12), DDR2 (1q23.3), NFASC (1q32.1), ESRRG (1q41), H3F3A (1q42.12), FH (1q43)
Chromosome 19
STK11 (19p13.3), GNA11 (19p13.3), MAP2K2 (19p13.3), SMARCA4 (19p13.2), JAK3 (19p13.11), CCNE1 (19q12), AKT2 (19q13.2), AXL (19q13.2), CIC (19q13.2), PPP2R1A (19q13.41)

Image analysis

PET images were obtained using Discovery PET/CT 710 (GE Healthcare, Milwaukee, WI, USA). The 11C-methionine tracer was injected intravenously at 185-280 mBq (5-7.5 mCi). The total activity, measured 20 min after tracer injection, was used for image reconstruction. The images were stored in anisotropic voxels (128 × 128 × 16; voxel size, 1 × 1 × 2.6 mm). The PET images were independently interpreted by two experienced nuclear physicians blinded to the clinical and anatomical imaging findings, and a consensus was reached. Tracer uptake by the lesion was evaluated using visual and semi-quantitative analyses. For semi-quantitative analysis, a region of interest (ROI) was placed over the entire lesion on the transverse PET image, and the standardized uptake value (SUV) was calculated. For lesions with reduced or equal tracer uptake, the ROI was selected based on anatomical information of brain lesions provided by magnetic resonance imaging or computed tomography (CT). A normal ROI was selected on the contralateral side of tumors of the same size. The tumor-to-normal tissue (T/N) ratio was calculated stereotactically by dividing the SUVmax of the tumor by that of the contralateral lesion.

18F was produced using an HM-12 cyclotron (Sumitomo Heavy Industries, Tokyo, Japan), and 18F-FDG was synthesized using an F-200 (Sumitomo Heavy Industries) with a radiochemical purity of > 95%. A dose of 180-220 MBq of 18F-FDG was intravenously administered to patients after fasting for at least 5 h and when their blood glucose levels were within the normal limit (6.4 mM). After 60 min, patients underwent 18F-FDG-PET/CT under standardized conditions. Overall, 299 axial slides with an inter-slice spacing of 3.27 mm were acquired. The PET images were interpreted independently by an experienced neurosurgeon. Tracer uptake by the lesions was evaluated as described for MET-PET. Visual emissions were also assessed.

Statistical analysis

Statistical analyses were performed using Prism 9 software (GraphPad Software Inc., California, USA). Fisher’s exact test and t-test were used to analyze data with a normal distribution. Patient survival was determined using the Kaplan-Meier method and Gehan-Breslow-Wilcoxon test. The receiver operating characteristic (ROC) curve was used to evaluate the diagnostic accuracy of MET-PET and FDG-PET, expressed as the area under the ROC curve (AUC). The cut-off values for PET scans were determined to maximize the sensitivity and specificity in distinguishing WHO grade 4 (GBM, IDH-wildtype, astrocytic glioma, and IDH-mutant) and grades 2-3 tumors (astrocytic glioma, IDH-mutant). P ≤ 0.05 was considered statistically significant.

## Results

MET and FDG uptake in astrocytic gliomas

The clinical characteristics of all 32 patients (age range, 25-85 years; 18 males and 14 females) are presented in Table 2. According to the WHO 2021 classification, IDH-wildtype astrocytic glioma is diagnosed as GBM, i.e., molecular GBM, if at least one of the following molecular features is present: TERTp mutation, EGFR amplification, or the combined whole chromosome 7 gain and whole chromosome 10 loss (chr 7+/10−). If none of these features are present, it is diagnosed as not elsewhere classified (NEC). The tumors identified in the study were classified as GBM, IDH wildtype (n = 15); astrocytic glioma, IDH-mutant, WHO grade 4 (astrocytic glioma G4) (n = 2); astrocytic glioma, IDH-mutant, WHO grade 3 (astrocytic glioma G3) (n = 4); astrocytic glioma, IDH-mutant, WHO grade 2 (astrocytic glioma G2) (n = 7); and NEC (n = 4).

**Table 2:** Clinical characteristics of the patient group based on the 2021 WHO classification (n = 32) IDH, isocitrate dehydrogenase; NEC, not elsewhere classified; FDG, fluorodeoxyglucose; MET, methionine; PET, positron emission tomography; SUVmax, maximum standardized uptake value; T/N ratio, the ratio of SUVmax in tumor to SUVmax in contrast normal tissue; MRI, magnetic resonance imaging

		All	Glioblastoma, IDH-wildtype	Astrocytoma, IDH-mutant, WHO grade 4	Astrocytoma, IDH-mutant, WHO grade 3	Astrocytoma, IDH-mutant, WHO grade 2	Diffuse astrocytoma, NEC
No. of cases		32	15	2	4	7	4
Age, years		55.3±16.2 (25-85)	61.9±15.3 (25-85)	43.5±0.5 (43-44)	50.0±17.0 (28-75)	47.7±13.3 (31-71)	55.0±16.3 (33-77)
Sex	Male	18	8	1	2	5	2
	Female	14	7	1	2	2	2
Ki-67 (%)		17±15	24±15	30±20	10±6	4±3	10±12
MET-PET	SUVmax	3.89±2.45	5.38±2.59	3.75±0.75	2.13±0.58	2.24±1.29	3.00±1.48
	T/N ratio	2.37±1.42	3.22±1.53	2.04±0.28	1.32±0.29	1.43±0.73	2.03±0.91
FDG-PET	SUVmax	7.87±2.56	8.69±2.54	9.42±0.20	6.21±0.56	5.72±1.55	9.48±2.56
	T/N ratio	0.77±0.32	0.85±0.32	1.01±0.33	0.48±0.17	0.58±0.16	0.98±0.21

**Figure 1:**
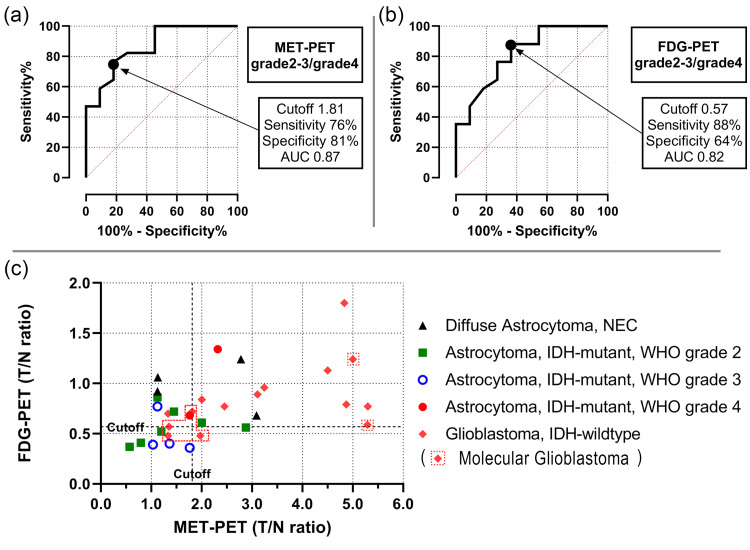
Cut-off values for MET-PET, FDG-PET, and imaging characteristics of all tumors (a) The optimal T/N ratio cut-off for MET-PET to distinguish between astrocytoma grades 2-3, astrocytoma grade 4, and glioblastoma was 1.81. (b) The optimal T/N ratio cut-off for FDG-PET to distinguish between astrocytoma grades 2-3, astrocytoma grade 4, and glioblastoma was 0.57. (c) A scatter plot presents the image characteristics of each patient with the T/N ratio of FDG-PET on the vertical axis and the T/N ratio of MET-PET on the horizontal axis. PET, positron emission tomography; MET, l-methyl-11C-methionine; 18F-FDG, fluorine-18 fluorodeoxyglucose; SUVmax, maximum standardized uptake value; T/N ratio, ratio of SUVmax in tumors to SUVmax in normal tissues

For 28 cases (excluding the NECs), the optimal T/N ratio cut-off for MET-PET to differentiate astrocytic glioma G2-3 from G4 and GBM was 1.81 (sensitivity, 76%; specificity, 81%; AUC, 0.87; Figure 1a). For FDG-PET, the optimal T/N cut-off was 0.57 (sensitivity, 88%; specificity, 64%; AUC 0.82; Figure 1b). Astrocytic glioma G2-3 had low MET and FDG uptake, while GBMs had high uptake of both (Figure 1c).

**Figure 2:**
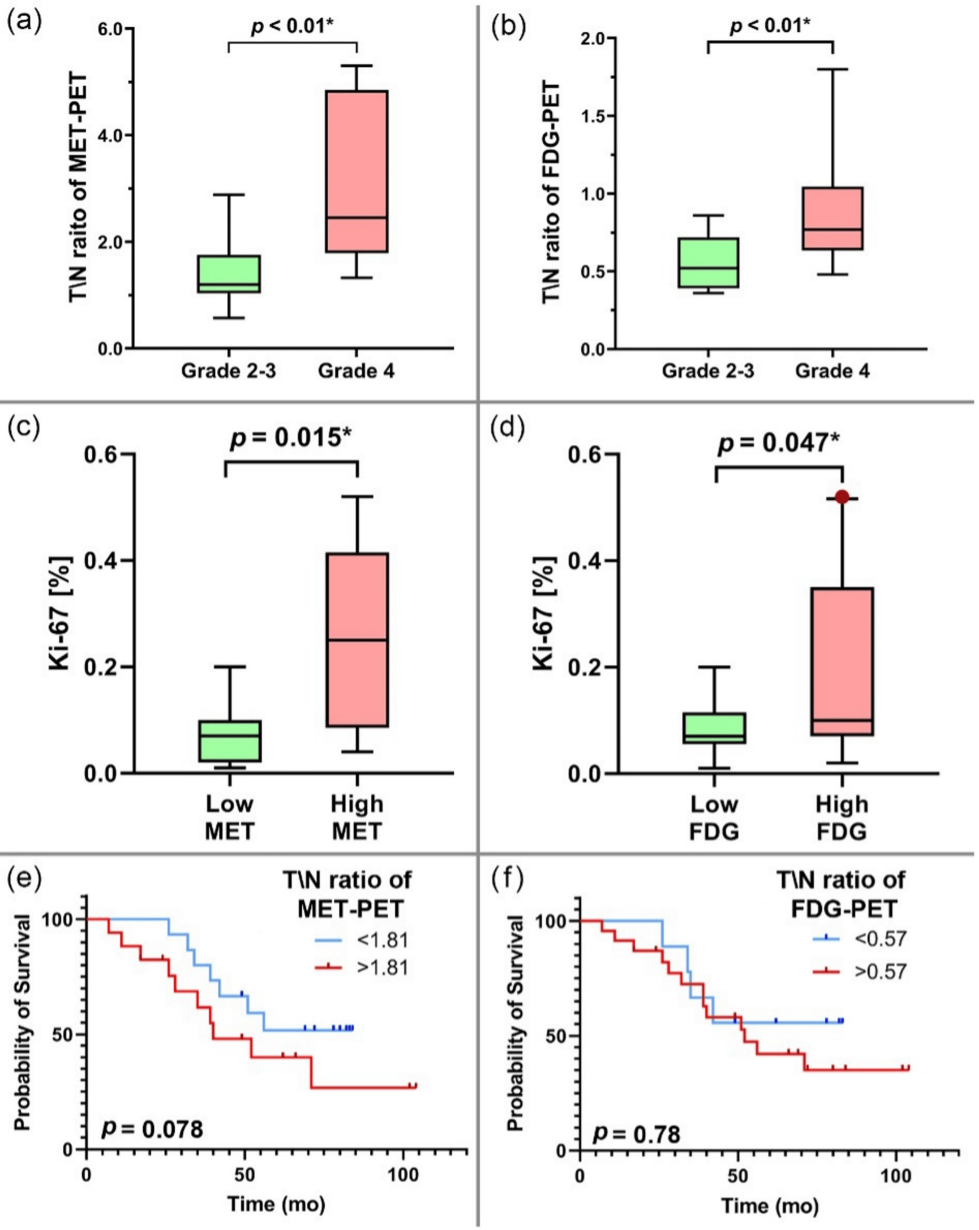
Analysis of the T/N ratio for PET tracers according to histological grade, Ki-67 index, and overall survival (a) A significant correlation between the histological grade and T/N ratios of MET-PET (p < 0.01). (b) A significant correlation between the histological grade and T/N ratios of FDG-PET (p < 0.01). (c) Astrocytic gliomas with high MET accumulation had a higher Ki-67 index (p = 0.015). (d) Astrocytic gliomas with high FDG accumulation had a higher Ki-67 index (p = 0.047). (e) Overall survival in patients with astrocytic gliomas and high MET accumulation was shorter than that in those with low MET accumulation (p = 0.078). (f) No significant difference was observed in overall survival based on the differences in FDG accumulation (p = 0.78). PET, positron emission tomography; MET, l-methyl-11C-methionine; FDG, fluorodeoxyglucose; T/N ratio, ratio of SUVmax in tumors to SUVmax in normal tissues

A significant correlation was observed between tumor grade and the T/N ratio in MET-PET and FDG-PET (p < 0.01; Figures 2a, 2b). NECs with CDKN2A/B HD and PDGFRA amplification exhibited high MET and FDG uptake, while NECs without driver gene alterations had low MET uptake (Table 3).

**Table 3:** Genetic and imaging information for diffuse glioma, not classified elsewhere (NEC) (N=4) IDH, isocitrate dehydrogenase; FDG, fluorodeoxyglucose; MET, methionine; NEC, not elsewhere classified, PET, positron emission tomography; SUVmax, maximum standardized uptake value; T/N ratio, the ratio of SUVmax in tumor to SUVmax in contrast normal tissue; MRI, magnetic resonance imaging

Age	Sex	Ki-67 (%)	T/N ratio of MET-PET	T/N ratio of FDG-PET	IDH	Genetic alterations characteristic of glioma
77	female	10%	3.09	0.68	wildtype	NF1 mutation, CDKN2A/B homozygous deletion
62	male	30%	2.78	1.24	wildtype	TP53 mutation, RB1 loss, PDGFRA amplification
48	male	2%	1.12	0.92	wildtype	None
33	female	2%	1.13	1.06	wildtype	None

Patients with astrocytic gliomas and high MET (> 1.81; p = 0.015; Figure 2c) or FDG accumulation (> 0.57; p = 0.047; Figure 2d) had a higher Ki-67 index as compared to those with low accumulation. Patients with astrocytic gliomas and high MET accumulation were associated with poorer overall survival than those with low MET accumulation (p = 0.078; Figure 2e), whereas no significant difference in survival was observed between those with high FDG accumulation and those with low FDG accumulation (p = 0.78; Figure 2f).

Relationship between gene analysis and MET or FDG uptake in astrocytic gliomas

**Figure 3:**
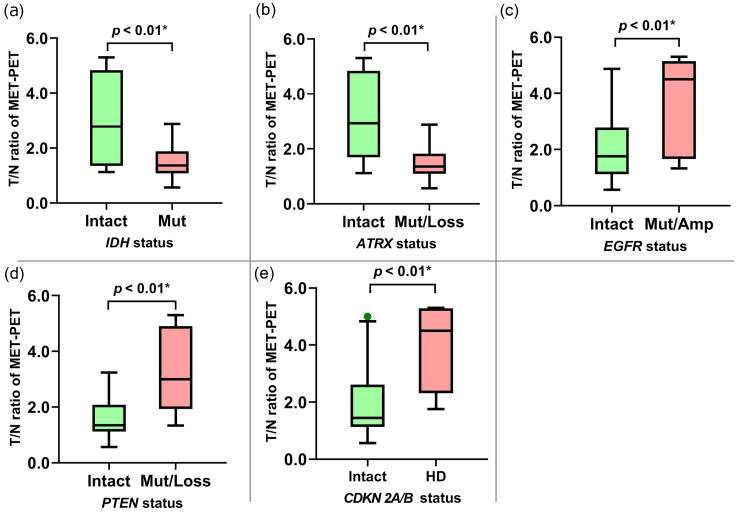
Relationship between gene alteration and MET accumulation in astrocytic gliomas (a) Astrocytic gliomas with IDH mutation had a significantly lower T/N ratio of MET-PET than those without. (b) Astrocytic gliomas with ATRX mut/loss had a significantly lower T/N ratio of MET-PET than those without. (c) Astrocytic gliomas with EGFR mut/amp had a significantly higher T/N ratio of MET-PET than those without. (d) Astrocytic gliomas with PTEN mut/loss had a significantly higher T/N ratio of MET-PET than those without. (e) Astrocytic gliomas with CDKN2A/B homozygous deletion had a significantly higher T/N ratio of MET-PET than those without. PET, positron emission tomography; MET, l-methyl-11C-methionine; MUT/AMP, mutation and/or amplification; MUT/LOSS, mutation and/or loss; SUVmax, maximum standardized uptake value; T/N ratio, ratio of SUVmax in tumors to SUVmax in normal tissues

**Figure 4:**
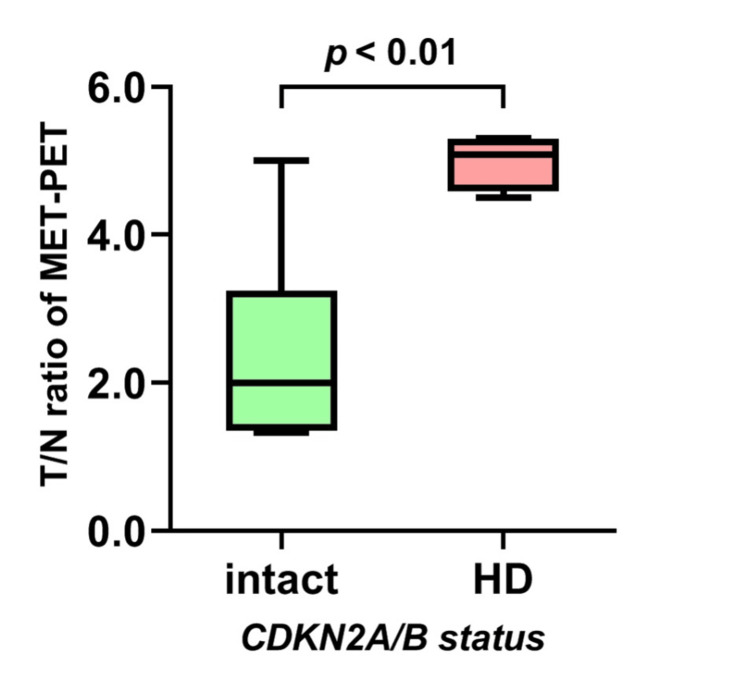
The T/N ratio for MET-PET in a focused investigation of GBM with CDKN2A/B HD In a focused investigation of GBM, the T/N ratio for MET-PET was significantly higher in patients with CDKN2A/B HD than in those without this deletion (p < 0.01).

The MET-PET T/N ratio was significantly lower in patients with astrocytic gliomas with IDH mutations (p < 0.01) or ATRX mutations and/or loss (mut/loss) (p < 0.01) compared with those without these alterations (Table 4, Figures 3a, 3b). The MET-PET T/N ratio was significantly higher in patients with EGFR mutations and/or amplifications (mut/amp) (p < 0.01), PTEN mut/loss (p < 0.01), and CDKN2A/B HD (p < 0.01) tumors compared with those without these alterations (Table 4, Figure 3c, 3e). A focused GBM investigation revealed a significantly higher MET-PET T/N ratio in patients with CDKN2A/B HD as compared with those without CDKN2A/B HD (p < 0.01; Figure 4).

**Table 4:** Relationship between PET tracers and genetic status in patients with astrocytic glioma The genetic alteration definitions are as follows: IDH1 mutation, ATRX mutation and/or loss, TP53 mutation and/or loss, TERT promoter mutation, NF1 mutation and/or loss, EGFR mutation and/or amplification, RB1 mutation and/or loss, PTEN mutation and/or loss, and CDKN2A/B homozygous deletion *p < 0.05, statistically significant PET, positron emission tomography

	MET-PET (T/N ratio)				FDG-PET (T/N ratio)				Ki-67			
	Alteration	Intact	t	p	Alteration	Intact	t	p	Alteration	Intact	t	p
IDH1	1.48±0.64	2.97±1.54	3.27	<0.01*	0.87±0.31	0.61±0.27	2.39	0.02*	10±13	21±16	2.04	0.05*
ATRX	1.49±0.62	3.04±1.54	3.52	<0.01*	0.58±0.17	0.91±0.34	3.26	<0.01*	9±8	23±17	2.8	<0.01*
TP53	2.02±1.06	2.81±1.76	1.56	0.12	0.75±0.38	0.79±0.23	0.36	0.71	19±18	14±12	-0.96	0.35
TERTp	2.87±1.78	2.14±1.24	-1.35	0.18	0.76±0.25	0.77±0.35	0.03	0.98	16±13	17±17	0.17	0.87
NF1	3.21±1.50	2.08±1.33	-1.99	0.06	0.96±0.42	0.70±0.26	-2.04	0.04*	25±18	14±14	-1.88	0.07
EGFR	3.50±1.77	1.92±1.02	-3.17	<0.01*	0.90±0.42	0.71±0.27	-1.46	0.15	19±14	16±16	-0.48	0.64
RB1	2.59±1.20	2.23±1.58	-0.68	0.5	0.90±0.34	0.68±0.28	-1.93	0.06	24±18	13±13	-2.04	0.05*
PTEN	3.36±1.51	1.59±0.77	-4.28	<0.01*	0.83±0.35	0.71±0.30	-1.05	0.29	24±16	11±12	-2.74	<0.01*
CDKN2A/B	3.87±1.46	1.94±1.14	-3.7	<0.01*	0.85±0.27	0.73±0.33	-0.77	0.44	27±15	14±15	-2.06	0.05*

The FDG-PET T/N ratio was significantly lower in patients with astrocytic gliomas with IDH mutations (p = 0.02) or ATRX mut/loss (p < 0.01) compared with those without these alterations (Table 4). Furthermore, the FDG-PET T/N ratio was significantly higher in patients with astrocytic gliomas with NF1 mut/loss (p = 0.04) compared with those without this alteration (Table 4). No significant difference was observed in the MET-PET T/N ratio (p = 0.18) or FDG-PET T/N ratio (p = 0.98) between patients with TERTp mutations and those without these mutations (Table 4).

The Ki-67 index was significantly lower in patients with astrocytic gliomas with IDH mutations (p = 0.05) and ATRX mut/loss (p < 0.01) compared with those without these alterations (Table 4). Furthermore, the Ki-67 index was significantly higher in patients with astrocytic gliomas with CDKN2A/B (p = 0.05), RB1 mut/loss (p = 0.05), and PTEN mut/loss (p < 0.01) compared with those without these alterations (Table 4).

**Figure 5:**
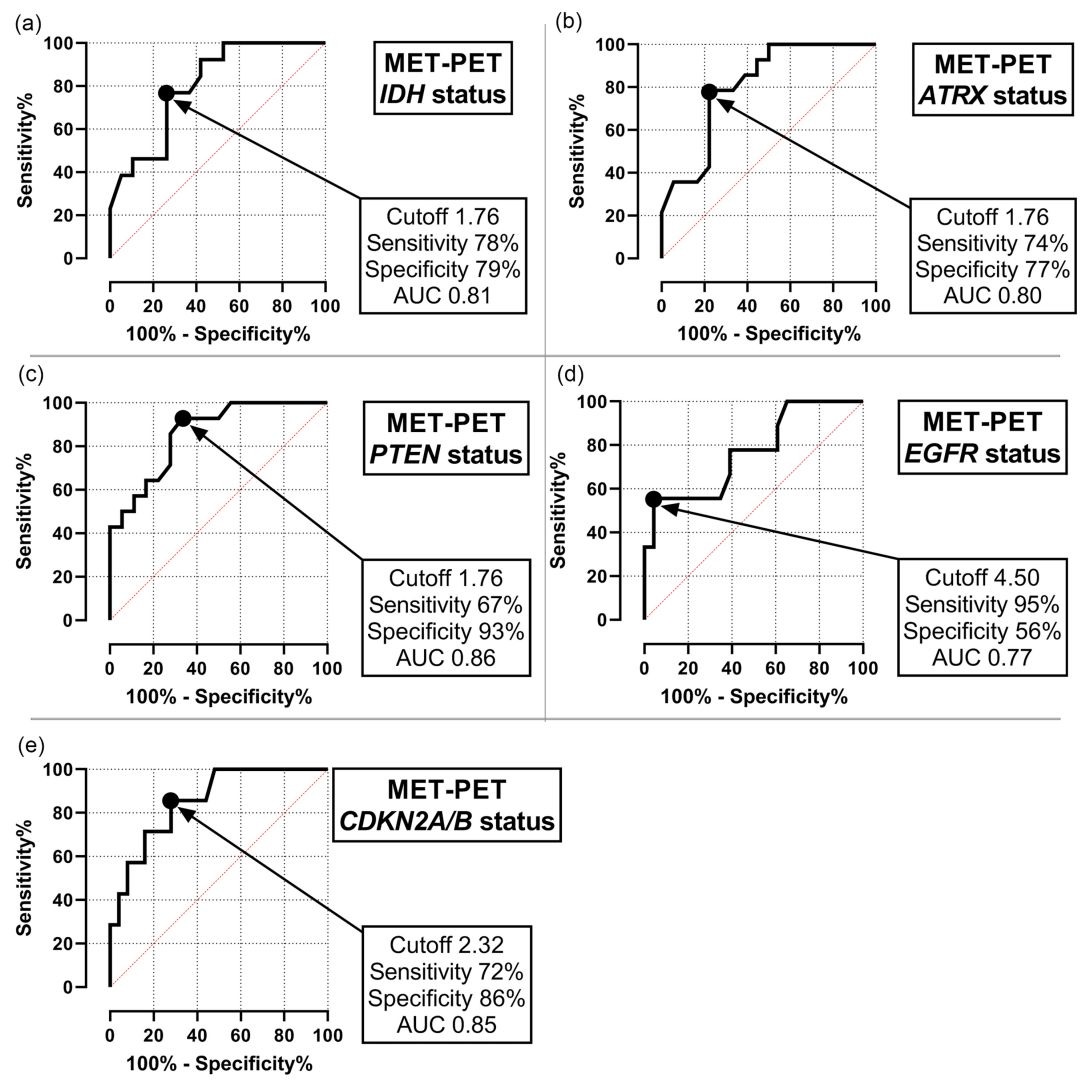
Appropriate cut-off values for T/N ratio of MET-PET identifying gene alterations in astrocytic gliomas (a) Cut-off value for identifying IDH mutation. (b) Cut-off value for identifying ATRX mut/loss. (c) Cut-off value for identifying PTEN mut/loss. (d) Cut-off value for identifying EGFR mut/amp. (e) Cut-off value for identifying CDKN2A/B homozygous deletion. PET, positron emission tomography; MET, l-methyl-11C-methionine; MUT/AMP, mutation and/or amplification; MUT/LOSS, mutation and/or loss; SUVmax, maximum standardized uptake value; T/N ratio, ratio of SUVmax in tumors to SUVmax in normal tissues

**Figure 6:**
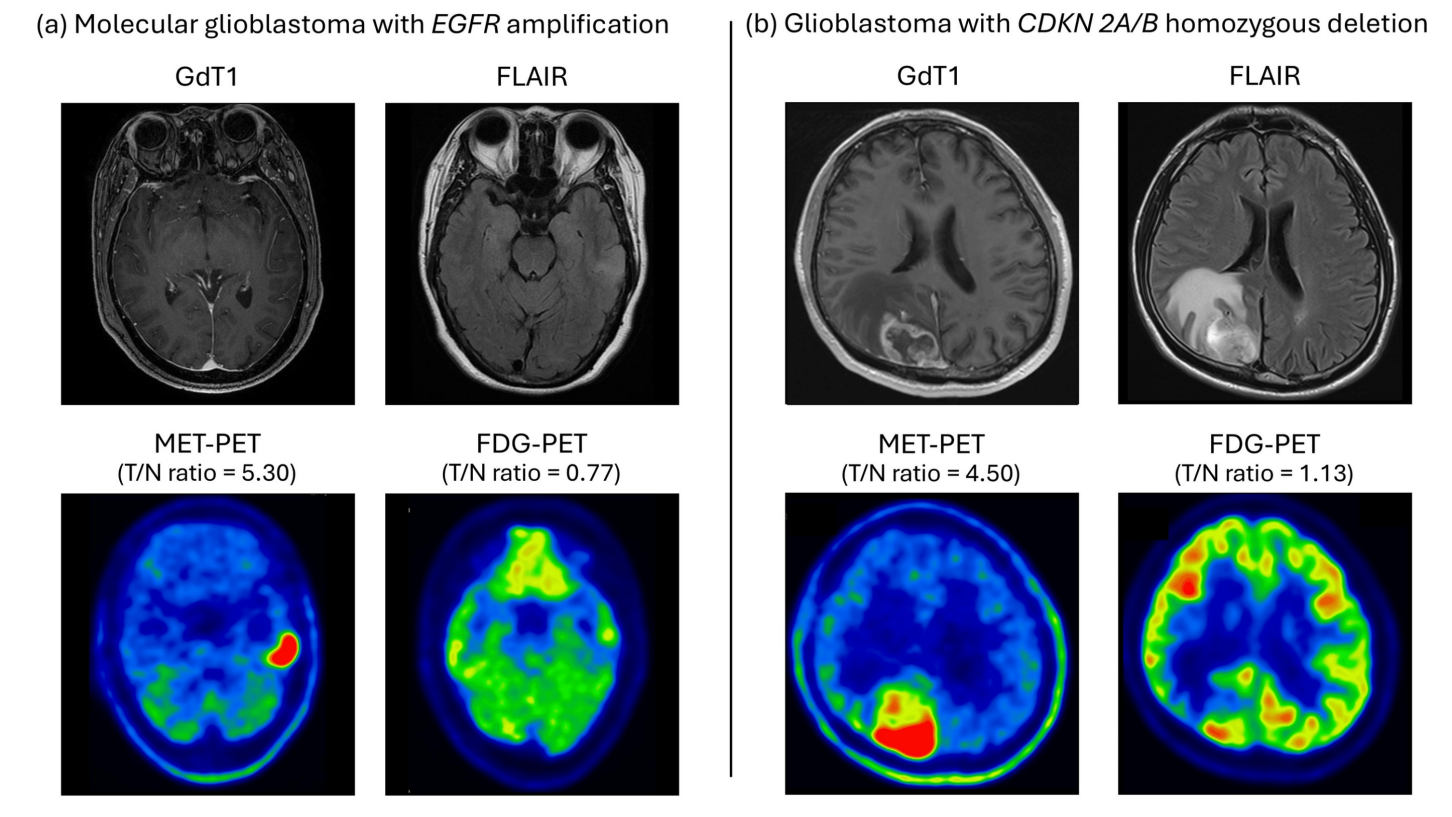
Representative cases of molecular glioblastoma with EGFR amplification and IDH-mutant, WHO grade 4 astrocytoma with CDKN2A/B homozygous deletion (HD) (a) The molecular glioblastoma case with the lesion observed in the left temporal lobe, no contrast enhancement on GdT1 MRI, hyperintensity on FLAIR, and reduced glucose uptake on FDG-PET (T/N ratio = 0.77). The lesion had intense methionine uptake on MET-PET (T/N ratio = 5.30). (b) The IDH-mutant, WHO grade 4 astrocytoma was associated with a mass lesion in the right occipital lobe, with enhancement on GdT1 MRI, peritumoral edema on FLAIR, increased metabolic activity on MET-PET (T/N ratio = 4.50), and reduced glucose uptake on FDG-PET (T/N ratio = 1.13). PET, positron emission tomography; MET, l-methyl-11C-methionine; 18F-FDG, fluorine-18 fluorodeoxyglucose; MRI, magnetic resonance imaging; FLAIR, fluid-attenuated inversion recovery; T/N ratio, SUVmax, maximum standardized uptake value; ratio of SUVmax in tumors to SUVmax in normal tissues Note: All images shown in this figure are from cases diagnosed and treated at the author's institution.

Subsequently, we estimated appropriate cut-off values of the MET-PET T/N ratio for the detection of the gene alterations associated with significant tracer uptake (IDH, ATRX, EGFR, PTEN, CDKN2A/B) using a ROC curve analysis (Figure 5). The cut-off value for the MET-PET T/N ratio for identifying IDH mutation, ATRX mut/loss, and PTEN mut/loss was 1.76 (Figures 5a-5c). For EGFR mut/amp, the cut-off value for the MET-PET T/N ratio was 4.50 (sensitivity: 95%; specificity: 56%, AUC: 0.77) (Figure 5d). Figure 6a presents the representative case of molecular GBM with EGFR amplification. Additionally, the cut-off value of the MET-PET T/N ratio for CDKN2A/B HD was estimated to be 2.32 (sensitivity, 72%; specificity, 86%; AUC, 0.85) (Figure 5e). Figure 6b presents the representative case of GBM with CDKN 2A/B HD.

## Discussion

This study evaluated the relationship between gene alterations in diffuse astrocytic gliomas and PET tracer accumulation. In astrocytic tumors, FDG and MET uptake correlated with the histological grade. We conducted molecular analysis of astrocytic tumors based on the WHO 2021 classification. Our findings revealed an association between IDH mutations, ATRX mut/loss, NF1 mut/loss, PTEN mut/loss, CDKN2A/B HD, and EGFR mut/amp with the MET-PET T/N ratio. Furthermore, we revealed an association between IDH mutations, ATRX mut/loss, and NF1 mut/loss with the FDG-PET T/N ratio. In particular, CDKN2A/B HD was associated with strong MET accumulation, including in GBMs.

In our study, genetic alterations that showed a strong correlation with MET uptake also exhibited a robust association with the Ki-67 index, more so than with FDG uptake. These findings suggest that MET uptake may more directly reflect tumor proliferative activity compared to FDG uptake [12].

IDH1 mutations exhibit lower MET and FDG accumulation, consistent with our findings [7]. A previous study suggested that IDH1 mutations result in the activation of HIF-1α and the normalization of aerobic glycolysis, leading to reduced FDG uptake [7]. Moreover, gliomas with ATRX loss have been reported to have lower MET-PET T/N ratios than those with wildtype ATRX [9], similar to the findings of this study. Furthermore, tumors with ATRX mutations had lower FDG accumulation compared with those without. Although no previous study has investigated the relationship between ATRX status and FDG uptake, ATRX function loss or mutations mainly occur in low-grade gliomas and are rare in high grade gliomas [13].

Consistent with the findings of previous studies, we found no significant association between TERTp mutation and PET tracer uptake in astrocytic gliomas. FDG-PET was found to be ineffective for predicting TERTp mutations in patients with IDH-wildtype low grade gliomas [14]. Diffuse gliomas with TERTp mutations demonstrated no significant differences in the T/N ratio on MET-PET images compared with those of TERTp-wildtype gliomas [10]. Diffuse gliomas with TERTp mutations demonstrated significantly different "mean" T/N ratios (calculated using the tumor SUVmean, unlike our metric, which uses SUVmax) on MET-PET images as compared with their wildtype counterparts, but this finding is likely attributable to the inclusion of oligodendrogliomas in their cohort, which are known to have both high MET uptake and abundant TERTp mutations [10]. TERTp mutations are known for their dual prognostic role in gliomas; they are a negative prognostic marker in IDH-wildtype gliomas but a favorable marker in IDH-mutant gliomas [10,15]. In prior studies on diffuse gliomas stratified by IDH mutation status, the differences in the T/N ratio associated with TERTp mutations were relatively minor compared to those associated with IDH status, and appeared to primarily reflect underlying histological subtypes [10].

This study also revealed a significant association between EGFR mut/amp or PTEN mut/loss and MET accumulation in astrocytic tumors. To the best of our knowledge, this association has not been reported previously. In gliomas with EGFR mut/amp, growth factor signaling can directly promote amino acid uptake and utilization through increased Akt activity and mTORC1 activation [16]. PTEN is also a direct negative regulator of the PI3K/AKT/mTOR pathway [17]. Akt and mTORC1 activation induce and sustain cell surface expression of amino acid transporters [16]. This may explain the increased amino acid uptake observed in GBM with EGFR mut/amp or PTEN mut/loss.

In the present study, increased FDG uptake was observed in gliomas with NF1 mut/loss. Although the exact mechanisms of glucose metabolism in astrocytic gliomas with NF1 mut/loss remain unclear, NF1 mutations in astrocytic gliomas may regulate glucose metabolism through Akt/mTOR activity and increased glucose transporter expression [18].

The MET-PET T/N ratio was significantly higher in patients with astrocytic tumors with CDKN2A/B HD as compared to those without HD. The CDKN2A gene encodes the proteins p14ARF and p16INK4A [19], and CDKN2B encodes p15INK4b [20]. These proteins inhibit cell cycle progression; therefore, CDKN2A/B HD may disrupt the regulatory mechanisms controlling tumor cell proliferation, potentially leading to increased protein synthesis and MET accumulation [21]. Furthermore, MTAP codeletion has been reported in tumors with CDKN2A/B HD [22,23]. MTAP, a key enzyme in the MET salvaging pathway, can affect MET metabolism in GBM [24,25]. In line with these observations, we observed a significant association between CDKN2A/B HD and MET accumulation in astrocytic tumors.

According to the WHO 2021 Classification of Gliomas, EGFR amplification and CDKN2A/B HD play crucial roles in integrated malignancy diagnosis [1]. Our findings suggest that MET-PET imaging contributes to a better preoperative diagnosis by enabling improved detection of EGFR amplification or CDKN2A/B HD, aiding in treatment strategy planning.

This study had several limitations. This was a retrospective study with a limited number of cases for each genetic mutation. Consequently, we were unable to evaluate the effects of coexisting genetic alterations. The influence of such combinations on PET tracer uptake is likely complex and not merely additive. Therefore, further research with a larger cohort, including multivariate analysis, is warranted to investigate this.

Nevertheless, to the best of our knowledge, this is the first study to investigate the relationship between MET- or FDG-PET and alterations in driver genes, including EGFR, PTEN, NF1, and CDKN2A/B in gliomas.

## Conclusions

The findings of our retrospective small cohort study suggest that in astrocytic tumors, PTEN mut/loss, EGFR mut/amp, and CDKN2A/B HD are strong MET accumulation factors, and NF1 mut/loss is a strong FDG accumulation factor. These results suggest that PET has the potential to serve as a non-invasive strategy for predicting specific genetic alterations, functioning as both a diagnostic biomarker and a predictor of the molecular profile. Multicenter prospective trials are essential to further research on this topic.
